# *Grifola frondosa* (Maitake) extract as natural antioxidant on emulsion-type pork sausages

**DOI:** 10.1016/j.fochx.2025.102655

**Published:** 2025-06-12

**Authors:** Soyoung Jang, Sanghun Park, Gyutae Park, Beobmo Ku, Minjun Kim, Jiwoo Kang, Sungkyun Oh, Hyodong Han, Sohee Kim, Hwayong Lee, Sol-Hee Lee, Jungseok Choi

**Affiliations:** a Department of Animal Science, Chungbuk National University, Cheongju 28644, Republic of Korea; b Department of Forest Science, Chungbuk National University, Cheongju 28644, Republic of Korea

**Keywords:** *Grifola frondosa*, Antioxidant activity, Emulsion-type pork sausage, Meat quality, Lipid oxidation

## Abstract

This study examined the potential of *Grifola frondosa* extract (GF) as a natural antioxidant and functional ingredient in emulsion-type pork sausages. GF demonstrated dose-dependent radical scavenging activity in DPPH and ABTS assays, with a concentration of 5 mg/mL achieving effects similar to those of l-ascorbic acid. Total phenolic and flavonoid contents were 34.83 mg GAE/g and 5.73 mg QE/g. HPLC-DAD analysis identified phenolic acids including gallic acid, *p*-hydroxybenzoic acid, vanillic acid, caffeic acid, and *p*-coumaric acid. Sausages with five treatments were prepared: a negative control, a positive control with 0.1 % ascorbic acid, and GF at concentrations of 0.03 %, 0.06 %, and 0.09 %. Addition of GF increased moisture content and pH, consequently enhancing water-holding capacity and reducing cooking loss. TBARS and VBN levels decreased during refrigerated storage, although VBN slightly increased at 0.09 % GF. The 0.06 % GF treatment improved quality and storage stability, thus presenting a promising natural additive for meat products.

## Highlights

• *Grifola frondosa* extract (GF) shows strong antioxidant activity in vitro.
• Phenolic acids in GF were identified by high-performance liquid chromatography.
• GF increased water holding capacity and reduced cooking loss in pork sausages.
• GF addition to sausages inhibited lipid oxidation and protein degradation.
• 0.06 % GF was optimal for enhancing sausage quality and storage stability.

## 1 Introduction

Emulsified meat products consist of a highly hydrated protein matrix formed by adding salt to minced meat, with fat, water, and additives uniformly dispersed (Peña-Saldarriaga et al., 2020). These products cook easily, offer excellent texture and flavor, have high sensory acceptability, and are rich sources of essential amino acids, protein, iron, zinc, and vitamin B (de Souza Paglarini et al., 2019). Nevertheless, emulsion-type pork sausages typically have a high fat content and contain substantial levels of oxidative mediators such as unsaturated lipids, metal catalysts, and heme pigments, rendering them susceptible to oxidative degradation during refrigerated storage (Contini et al., 2014; Falowo et al., 2014).

Such lipid oxidation results in multiple issues such as discoloration, off-flavor production, formation of toxic compounds, proliferation of pathogenic microorganisms, reduced shelf life, and nutrient loss (Ayari et al., 2016; Wang et al., 2023). Consequently, synthetic antioxidants like butylated hydroxytoluene (BHT), butylated hydroxyanisole (BHA), tert-butylhydroquinone, and propyl gallate are frequently incorporated during meat product processing to mitigate these effects (Bellucci et al., 2022; Seo et al., 2021). However, the potential health risks linked to the long-term consumption of synthetic antioxidants have led to growing consumer interest in natural alternatives (Kumari et al., 2019; Manessis et al., 2020). Numerous studies have confirmed that the antioxidant properties of various fruits, vegetables, and herbs are largely attributable to their phenolic content. Phenolic compounds, among plant secondary metabolites, have frequently been shown to possess stronger antioxidant effects than synthetic antioxidants such as BHT and BHA (Wang et al., 2021).

*Grifola frondosa* (*G. frondosa*), a basidiomycete mushroom of the Polyporaceae family, is distinguished by its brown, wavy appearance and is known for its nutritional and pharmacological properties (Zhang et al., 2022). Medicinal mushrooms are abundant in crucial antioxidant compounds including phenolic acids, ergothioneine, flavonoids, and glutathione (Liuzzi et al., 2023). These antioxidants have been shown to attenuate lipid peroxidation and enhance the activity of antioxidant enzymes (Kalaras et al., 2017; Fulgoni III & Agarwal, 2021). Notably, *G. frondosa* is rich in trehalose, 5′-nucleotides, glutamic and aspartic acids, which impart a robust umami flavor (Spano et al., 2024). Consequently, it is utilized not only as a functional health ingredient but also as a flavor enhancer in various foods (Huang et al., 2011; Wu et al., 2021). Prior studies have primarily concentrated on the cultivation techniques and biological activities of this mushroom, including its antidiabetic, antitumor, anti-inflammatory, and antiallergic properties (Chien et al., 2017; He et al., 2019; Hetland et al., 2020), with fewer studies addressing its antioxidant potential. Therefore, the present study aims to assess the antioxidant activity of *G. frondosa* extract (GF) and investigate its effects on the quality and storage stability of emulsion-type pork sausages, in response to the growing demand for natural additives that can replace synthetic antioxidants in meat products.

## 2 Materials and methods

### 2.1 Preparation of *G. frondosa* extract

Dried fruiting bodies of *G. frondosa* (GRIFOLAN, batch no. [4845240302], Korea) were used. The samples were processed by adding 20 volumes of triple-distilled water and heated at 70 °C in a water bath (BS2–20 Shaking Heating Bath, JEIOTECH, Korea) for 3 h. The mixture was then filtered using Whatman No. 2 filter paper to remove residues. Subsequently, the filtrate was centrifuged at 13,000*g* for 15 min (Supra R17, Hanil Co., Korea), and the supernatant collected. The supernatant was freeze-dried with a freeze dryer (FDU-2100, EYELA, Japan) and stored at −20 °C for experimental use. The GF used in this study was obtained from a single batch of dried *G.frondosa* to ensure consistency across treatments. While this approach reduced sample variability, it may limit generalizability. Future studies should test multiple batches.

### 2.2 Analysis of antioxidant activities in *G. frondosa* extract

#### 2.2.1 2,2-Diphenyl-2-picrylhydrazyl (DPPH) radical scavenging assay

The DPPH radical scavenging assay was conducted by adapting the method developed by Blois (1958). GF was diluted with distilled water to achieve final concentrations of 1, 2, 3, 4, and 5 mg/mL. One milliliter of the sample was combined with 4 mL of 0.2 mM DPPH solution diluted in methanol, followed by incubation in the dark for 30 min. The absorbance was subsequently measured at 517 nm using a spectrophotometer, and the DPPH radical scavenging activity was expressed as a percentage, reflecting the difference in absorbance between the control and the sample.

$$\%\text{Inhibition}=\frac{\text{Absorbance}\;\left(\text{blank}\right)-\text{Absorbance}\;\left(\text{sample}\right)}{\text{Absorbance}\;\left(\text{sample}\right)}\times 100$$

#### 2.2.2 2,2′-Azino-bis-3-ethylbenzothiazoline-6-sulfonic acid (ABTS) assay

The ABTS radical scavenging assay was conducted by adapting the method of Re et al. (1999). GF was diluted with distilled water to achieve final concentrations of 1, 2, 3, 4, and 5 mg/mL. A solution of 7 mM ABTS and 2.45 mM potassium persulfate, mixed in a 1:1 ratio, was allowed to react in the dark at room temperature for 24 h to produce cations. Subsequently, the absorbance was adjusted to 0.70 ± 0.02 at 734 nm. The sample and ABTS radical solution were combined at a 1:19 ratio and incubated in the dark for 6 min before the absorbance was measured at 734 nm using a spectrophotometer. The ABTS radical scavenging activity was calculated as a percentage, based on the difference in absorbance between the control and the sample solution.

$$\%\text{Inhibition}=\frac{\text{Absorbance}\;\left(\text{blank}\right)-\text{Absorbance}\;\left(\text{sample}\right)}{\text{Absorbance}\;\left(\text{sample}\right)}\times 100$$

#### 2.2.3 Determination of total phenolic content (TPC) and total flavonoid content (TFC)

TPC was determined using the Folin-Ciocalteu reagent, following the protocol described by Forsido et al. (2013). A 1 mL aliquot of GF was combined with 5 mL of 0.2 N Folin-Ciocalteu reagent and allowed to react for 5 min. Subsequently, 4 mL of 7.5 % Na₂CO₃ was added, and the reaction mixture was incubated in the dark for 1 h. The absorbance was then measured at 765 nm using a spectrophotometer (Mobi, Microdigital, Korea). A standard curve, prepared using gallic acid, facilitated expression of the results as mg GAE/g (GAE: gallic acid equivalents).

TFC was determined following the method described by Singh et al. (2012). A 1 mL sample of GF was diluted fivefold with distilled water, and 0.3 mL of 5 % sodium nitrate solution was added. After 5 min, an equal volume of 10 % aluminum chloride was added and the mixture was homogenized using a vortex mixer (Labtron, VM1, Korea). The solution stood in the dark at room temperature for 5 min before the addition of 2 mL of 4 % sodium hydroxide. The absorbance of the reaction mixture was measured at 510 nm using a spectrophotometer. A standard curve was prepared using naringenin, and the results were expressed in mg QE/g (QE: quercetin equivalents).

#### 2.2.4 Phenolic acids analysis by HPLC-DAD

HPLC system chromatographic analysis was conducted using a YL 9100 (Young-Lin, Korea) equipped with a YL9101 vacuum degasser, YL9110 quaternary pump, YL9131 column compartment, and YL9120 UV/Vis detector. An Eclipse XDB C18 analytical column (4.6 mm × 150 mm, 5 μm) from Agilent, Santa Clara, USA, was maintained at 30 °C. Separations were conducted under isocratic conditions with a flow rate of 1.0 mL/min. The mobile phase was composed of acetonitrile (solvent A), an acetic acid solution adjusted to pH 3.0 (solvent B), and methanol (solvent C). The gradient elution program proceeded as follows: starting with 5 % A and 95 % B at 0 min, changing to 10 % A, 80 % B, and 10 % C at 10 min, then to 20 % A, 60 % B, and 20 % C at 20 min, and finishing with 100 % A at 30 min (Jahan et al., 2014). UV detection was carried out at wavelengths of 280 nm.

### 2.3 Manufacture of emulsion-type pork sausages

Pork hind leg meat (semimembranosus) and back fat for this study were purchased from local markets in Cheongju, Korea, approximately 24 h post-mortem. The pork was denuded of connective tissues and then minced using a silent bowl cutter (KC-B47, KCPACK, Korea). The experimental groups were established as follows: a negative control without additives (NC), a positive control containing 0.1 % l-ascorbic acid (PC), and treatment groups receiving GF at concentrations of 0.03 % (GF3), 0.06 % (GF6), and 0.09 % (GF9). GF concentrations were selected based on preliminary tests to avoid sensory deterioration observed at higher levels. To minimize variability, all meat was obtained from a single batch, thoroughly mixed for homogeneity, and randomly allocated to each treatment group.To facilitate extraction of salt-soluble proteins, 1.2 % salt and 1 % sugar were initially blended with the minced meat using a bowl cutter equipped with 4 knives for 2 min, followed by additional grinding. The minced meat was then combined with back fat, ice, *l*-ascorbic acid, and GF according to the specifications in Table 1, and further chopped for 2 min. Throughout this process, the initial meat temperature was maintained below 5 °C, and the final emulsion temperature was controlled below 11 °C. The emulsified meat mixtures were encased in 26 mm diameter collagen casings (NDX, VISCOFAN CZ, Czech), using a sausage stuffer (EM-12, Mainca, Spain), producing sausages approximately 13 cm in length. These sausages were then cooked in a water bath at 80 °C for 20 min. Post-cooking, the sausages were vacuum-packed using a vacuum packing machine (CV-400S, Hanato, Korea) and preserved at 4 °C for 3 weeks and utilized as samples for experiments. Packaged samples were stored in vacuum bags (LUWCOPACK, 4827232406, Korea) made of nylon with a thickness of 70 μm and having an O_2_ permeability <30 cc/m^2^ in 24 h. At each sampling time, individually packaged samples were opened to prevent repeated exposure to air.

**Table 1:** Formulations (%) of emulsion-type pork sausages with different levels of *G. frondosa* extract.

Ingredients (%)		Treatments				
		NC	PC	GF3	GF6	GF9
Main	Meat	60	60	60	60	60
	Back fat	20	20	20	20	20
	Ice	20	20	20	20	20
Additives	Sugar	1	1	1	1	1
	Salt	1.2	1.2	1.2	1.2	1.2
	l-ascorbic acid	–	0.1	–	–	–
	GF^1^	–	–	0.03	0.06	0.09

1 *G. frondosa* (Maitake) extract.

### 2.4 Analysis of emulsion-type pork sausages

#### 2.4.1 Proximate composition

Proximate composition analysis was performed following the protocol established by the Association of the Official Analytical Chemists (AOAC, 2012). Moisture content was determined using the atmospheric pressure drying method at 105 °C, while crude protein content was assessed utilizing the Kjeldahl method. Crude fat was extracted utilizing the Folch method, and crude ash content was determined via the direct ashing method.

#### 2.4.2 pH

To ascertain the pH value, 5 g of sausage sample was combined with 45 mL of distilled water and homogenized for 30 s using a homogenizer (Stomacher 400 Circulator lab blender, Seward, UK). The pH was then measured using a pH meter equipped with a pH electrode (Orion Star™ A211, Thermo Scientific, USA), which was calibrated with buffer solutions at pH 4.01, 7.00, and 10.01.

#### 2.4.3 Water holding capacity (WHC)

0.5 g of sausage sample was placed in the upper filter section of a centrifuge filter tube (Ultrafree Centrifugal Filter, Millipore Sigma, USA). The filter tube was then exposed to a water bath at 80 °C for 20 min. After heating, the upper filter section was assembled with the lower compartment and subjected to centrifugation at 3000 rpm for 10 min. Following centrifugation, the filter tube was removed. WHC (%) was ascertained by recording the weight differences before and after centrifugation.

#### 2.4.4 Cooking loss (CL)

To determine CL of the sausage, the sample was cut into cube-shaped pieces weighing approximately 50 g, vacuum-packaged in a sterilization bag (Ultraclean Sterilization Bag, KNF CRP, Korea), and then cooked in a water bath at 70 °C for 30 min. The CL percentage was calculated by determining the weight difference before and after cooking, and dividing it by the initial weight.

$$\text{Cooking loss}\;\left(\%\right)=\frac{\left(\text{Weight before cooking}-\text{Weight after cooking}\right)}{\text{Weight before cooking}}\times 100$$

#### 2.4.5 2-thiobarbituric and reactive substances (TBARS) values

The oxidative stability of sausages was assessed using the TBARS method as described by Tarladgis et al. (1960). A 5 g sample was mixed with 15 mL of distilled water and 50 μL of 7.2 % BHT, then homogenized for 30 s. Subsequently, 2 mL of TBA/TCA solution (20 mM TBA in 15 % TCA) was added to 1 mL of the homogenate, and the mixture was vortexed. The mixture was incubated in a water bath at 90 °C for 30 min, then cooled in cold water for 10 min, and centrifuged at 3000 rpm for 15 min at 4 °C. The absorbance of the resulting supernatant was measured at 532 nm, and the TBARS value was calculated as mg of malondialdehyde (MDA)/kg.

#### 2.4.6 Volatile basic nitrogen (VBN)

To quantify the extent of protein degradation, the procedure for measuring the VBN content (mg%) was performed according to Pearson (1968). A 10 g sample was combined with 90 mL of distilled water and homogenized at 10,000 rpm for about 30 s. The resultant mixture was subsequently filtered through Whatman No. 2 filter paper. A 1 mL aliquot of the filtrate was transferred to the outer chamber of a Conway unit, while 1 mL of 0.01 N boric acid solution, along with three drops of indicator solution (0.066 % methyl red +0.066 % bromocresol green), were added to the inner chamber. White Vaseline was applied around the lid's seal before it was secured. Subsequently, 1 mL of 50 % K₂CO₃ was introduced to the outer chamber; the unit was immediately sealed, gently agitated, and incubated at 37 °C for 2 h. Post-incubation, the boric acid solution in the inner chamber was titrated with 0.02 N H₂SO₄. The volatile basic nitrogen (VBN) content was quantified and expressed in terms of mg per 100 g of the sample.

$$\mathrm{VBN}\left(\frac{\mathrm{mg}}{100g}\right)=\frac{\left(a-b\right)\times F\times 28.014\times 100}{\text{amount of sample}\;\left(g\right)}$$

a: the amount of H_2_SO_4_ injected into the sample (mL)
b: the amount of H_2_SO_4_ injected into the blank (mL)
F: 0.02 N H_2_SO_4_ standardized index
28.014: amount of N required to titrate 1 mL of 0.02 N H_2_SO_4_

### 2.5 Statistical analysis

All sausages samples were prepared using a single batch of GF. The experimental results were analyzed using at least five repetitions, and all statistical analyses were performed using SPSS (26.0). A one-way ANOVA was conducted to evaluate the significance either among the treatment groups at each storage day or among storage periods within each treatment, though more advanced models and batch replication should be considered in future studies. Duncan's multiple range test (*P* < 0.05) was subsequently applied to compare the means and standard deviations.

## 3 Results and discussion

### 3.1 Antioxidant properties of *Grifola frondosa* extract

**Fig. 1:**
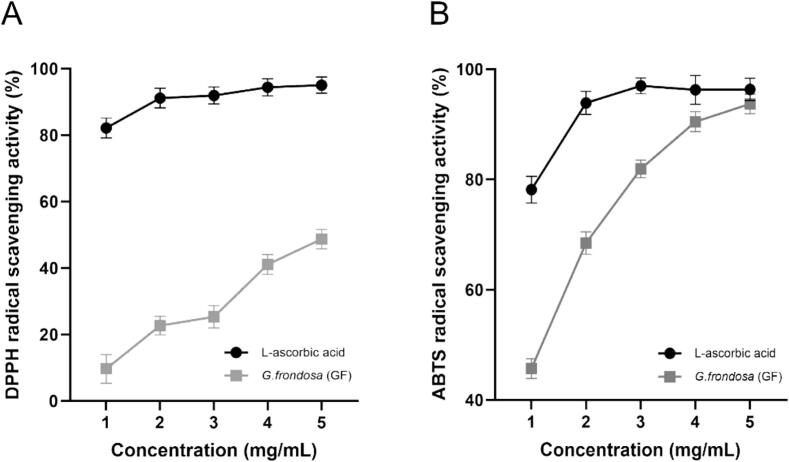
Antioxidant activities of *G. frondosa* extract: (A)DPPH radical scavenging activity and (B)ABTS radical scavenging activity.

The results of the DPPH and ABTS radical scavenging activities of GF are presented in Fig. 1. DPPH and ABTS are stable free radicals that assess the antioxidant capacity of substances by reducing their colored oxidized forms (Munteanu & Apetrei, 2021). In this study, l-ascorbic acid served as a positive control for comparison with GF. The DPPH radical scavenging activity of GF, at concentrations ranging from 1 to 5 mg/mL, varied from 9.66 % to 48.10 %, exhibiting a concentration-dependent increase. The ABTS radical scavenging activity ranged from 45.92 % to 93.30 %, and at a concentration of 5 mg/mL, it reached a value comparable to that of l-ascorbic acid. ABTS^•+^ contains polar functional groups such as –SO₃^−^ and non-polar components like a benzene ring, which enhances its solubility in both water and organic solvents, making it apt for detecting both hydrophilic and lipophilic antioxidants (Magalhães et al., 2008). In contrast, DPPH^•^ contains phenyl rings and –NO₂ groups, enhancing its solubility in organic solvents such as ethanol, which predominantly detects lipophilic antioxidants (Fernandes & Coimbra, 2023; Oboh et al., 2009). Therefore, the higher radical scavenging activity observed in ABTS^•+^ may be attributed to its efficacy in detecting a broader range of antioxidants, including water-soluble compounds. Overall, GF exhibits significant antioxidant activity by neutralizing free radicals and demonstrates potential as a beneficial antioxidant component, especially in hydrophilic environments.

The results of TPC and TFC in GF are presented in Table 2. These compounds can be structurally classified into phenolic acids, which feature a carboxylic acid group (–COOH) attached to a benzene ring, and flavonoids, characterized by a C6-C3-C6 benzene ring structure (Olszowy, 2019). The TPC and TFC of GF were determined to be 38.83 mg GAE/g and 5.73 mg QE/g, respectively. Polyphenols, significant natural antioxidants in many edible mushrooms, are compounds that feature one or more hydroxyl groups (–OH) and display strong correlations with antioxidant activity (Azieana et al., 2017). Generally, mushrooms are recognized for containing phenolic acids as their primary antioxidant compounds, while their flavonoid content is usually low (Heleno et al., 2010). These results align with the previous findings that the TPC of *G. frondosa* extracts exceeds the TFC (Angelova et al., 2022; Islam et al., 2016). The TPC and TFC values obtained in this study surpassed those previously recorded for *Pleurotus ostreatus* (19.37 mg GAE/g and 2.71 mg Rutin (RU)/g, respectively) and *Lentinula edodes* (24.14 mg GAE/g and 5.79 mg RU/g). They also exceeded those reported for *Flammulina velutipes* (enoki mushroom), with TPC and TFC values of 12.44 mg GAE/g and 2.41 mg QE/g, respectively (Elhusseiny et al., 2021; Hong et al., 2022). These findings suggest that GF may exhibit superior antioxidant activity compared to other mushrooms, primarily due to its high phenolic acid content.

**Table 2:** Total phenolic content (TPC) and total flavonoid content (TFC) of *G. frondosa* extract.

Parameters	Values
TPC (mg GAE^1^/g)	34.83 ± 0.47
TFC (mg QE^2^/g)	5.73 ± 0.38

1 GAE, gallic acid equivalents.

2 QE, quercetin equivalents.

### 3.2 Identification of phenolic compounds of *Grifola frondosa* extract

**Fig. 2:**
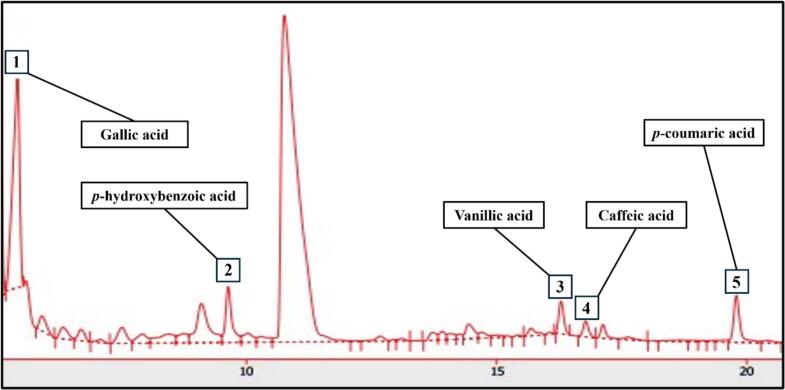
Representative HPLC chromatogram of phenolic compounds of *G. frondosa* extract recorded at 280 nm.Peaks: (1) gallic acid; (2) *p*-hydroxybenzoic acid; (3) vanillic acid; (4) caffeic acid; (5) *p*-coumaric acid.

Based on the results mentioned above, the phenolic acid content in GF was quantified using HPLC-DAD, as detailed in Table 3. Each compound was identified by comparing its retention time (RT) with that of standard phenolic acids in the chromatogram, and the results were expressed in mg per kg of dry extract (Fig. 2). Phenolic acids are recognized as secondary metabolites derived from the pentose phosphate, shikimate, and phenylpropanoid pathways in plants (Heleno et al., 2012). These compounds have been documented to exhibit a range of biological activities, including antioxidant, anti-inflammatory, antibacterial, antifungal, and anti-HIV effects (Çayan et al., 2020; Hu et al., 2016). Five phenolic acids were identified in GF, with gallic acid displaying the highest concentration at 223.03 mg/kg, followed by *p*-hydroxybenzoic acid (21.36 mg/kg), vanillic acid (25.26 mg/kg), caffeic acid (9.26 mg/kg), and *p*-coumaric acid (20.67 mg/kg). These concentrations are higher than those recorded for *Boletus edulis*, a mushroom known for its potent antioxidant activity, where gallic acid was quantified at 212.96 μg/g (Palacios et al., 2011), and more than twice the levels of caffeic acid (3.91 mg/kg) and *p*-coumaric acid (9.57 mg/kg) found in *Pleurotus ostreatus* (Dilfy et al., 2020). Although the *p*-hydroxybenzoic acid content in GF was marginally lower than the 25.59 mg/kg reported for *Agaricus bisporus*, the most widely consumed edible mushroom worldwide, vanillic acid and p-coumaric acid, absent in *A. bisporus*, were detected in GF (Barros et al., 2009). These results indicate that the antioxidant compounds in GF may enhance the oxidative stability and extend the shelf life of meat products. Accordingly, emulsion-type pork sausages were formulated with the addition of GF extract to assess its impact on quality and storage characteristics.

**Table 3:** Phenolic compounds of *G. frondosa* extract.

Phenolic compounds	RT (min)	Content (mg/kg of dry extract)
Gallic acid	5.43	223.03 ± 4.59
*p*-Hydroxybenzoic acid	9.64	21.36 ± 0.45
Vanillic acid	16.29	25.26 ± 0.33
Caffeic acid	16.78	9.26 ± 0.10
*p*-Coumaric acid	19.79	20.67 ± 1.11

### 3.3 Proximate composition of sausages

The proximate composition of emulsion-type pork sausages supplemented with varying levels of GF is detailed in Table 4. The moisture content was higher in the GF3, GF6, and GF9 samples compared to the NC and PC groups (*P* < 0.05). Hot water extracts of mushrooms are rich in hydrophilic polysaccharides such as β-glucan, α-glucan, and chitin, which possess numerous –OH groups capable of forming hydrogen bonds with water molecules, thus enhancing the WHC (Tu et al., 2021). This phenomenon may have contributed to the observed increase in the moisture content of the meat products. Crude protein content appeared unchanged by the addition of GF, likely owing to fluctuations in moisture and other variables (Parimelazhagan & Thangaraj, 2016). Both the crude fat and ash contents exhibited an upward trend with increased levels of GF supplementation (*P* < 0.05). The rise in crude fat content is likely derived from the intramuscular fat of the raw meat and other additives, whereas the increment in ash content is probably due to mineral elements such as magnesium, sodium, calcium, and iron, which are residues post-incineration (Iswoyo et al., 2022). The fruiting body of *Grifola frondosa* contains approximately 6–7 % lipids, predominantly unsaturated fatty acids, and is also rich in minerals such as calcium, sodium, potassium, and iron (Wang et al., 2012; Negi et al., 2024). Consequently, the slight increases observed in crude fat and ash contents could be attributed to the unsaturated fatty acids and mineral components present in GF. Slight variations in total proximate composition may result from inherent variability in raw materials, processing conditions, and analytical procedures as commonly observed in commercial meat products (Jiménez-Colmenero et al., 2010).

**Table 4:** Proximate composition of emulsion-type pork sausages with *G. frondosa* extract powder.

Parameters	Treatments				
	NC	PC	GF3	GF6	GF9
Moisture	57.16 ± 0.91^b^	57.49 ± 0.09^b^	61.80 ± 0.08^a^	62.15 ± 0.65^a^	62.47 ± 0.35^a^
Crude protein	16.78 ± 0.52^bc^	17.61 ± 0.62^a^	17.09 ± 0.30^ab^	17.38 ± 0.29^a^	16.42 ± 0.12^c^
Crude fat	20.73 ± 0.74^a^	19.87 ± 0.45^ab^	16.48 ± 1.02^d^	17.32 ± 0.50^cd^	18.80 ± 1.16^bc^
Crude ash	2.11 ± 0.06^ab^	2.08 ± 0.05^b^	2.12 ± 0.07^ab^	2.14 ± 0.05^ab^	2.21 ± 0.06^a^

^a-d^ Different letters within each row indicate significant differences determined by mean ± standard devision (*P* < 0.05).

Treatments: NC, no addition; PC, 0.1 % *l*-ascorbic acid; GF3, 0.03 % GF; GF6, with 0.06 % GF; GF9, 0.09 % GF.

### 3.4 pH, water holding capacity and cooking loss of sausages

Moisture loss in meat products can lead to reduced final product weight and economic losses (Cheng & Sun, 2008). Consequently, moisture content is regarded as a critical quality parameter in the meat processing industry due to its impact on yield (Bertram et al., 2003). Generally, high WHC and low CL in meat products are valued as indicators of quality from both nutritional and economic viewpoints (Pathare & Roskilly, 2016). The pH, WHC, and CL of emulsion-type pork sausages supplemented with different levels of GF are presented in Table 5. The pH was lowest in the PC group and increased with higher levels of GF supplementation (*P* < 0.05). These results align with the findings of Choe et al. (2018), who observed that incorporating winter mushroom powder as a phosphate replacer in emulsified sausages elevated the pH. When meat pH exceeds the isoelectric point of proteins (5.0–5.2), the net negative charge on myofibrillar proteins increases, consequently reducing water and fat release (Bertram et al., 2004). The WHC was highest in the GF6 and GF9 groups, and CL was lowest in these groups (*P* < 0.05). The amino acid profile of GF, as reported by An et al. (2020), includes high levels of basic amino acids such as arginine and histidine, and polar amino acids like threonine, tyrosine, and cystine. Basic amino acids can elevate pH by accepting hydrogen ions, while hydrophilic polar amino acids and polysaccharides featuring –OH groups form hydrogen bonds with water molecules, thus stabilizing protein structures and enhancing water retention (Bender, 2012; Tu et al., 2021). Thus, it is presumed that the amino acid composition and hydrophilic polysaccharides present in GF contributed to the increased pH and stabilized protein structures, leading to improved water retention. As a result, the observed reduction in CL in this study is likely due to increased WHC, and the inclusion of GF appears to improve the overall quality characteristics of the sausages.

**Table 5:** pH, water holding capacity (WHC), and cooking loss (CL) of emulsion-type pork sausages with *G. frondosa* extract powder.

Parameters	Treatments				
	NC	PC	GF3	GF6	GF9
pH	5.87 ± 0.01^d^	5.78 ± 0.00^e^	5.92 ± 0.02^c^	5.94 ± 0.00^b^	5.96 ± 0.00^a^
WHC (%)	58.91 ± 1.60^b^	50.64 ± 2.69^c^	64.59 ± 3.95^ab^	66.86 ± 2.63^a^	66.93 ± 4.78^a^
CL (%)	40.92 ± 2.55^a^	39.32 ± 0.99^a^	33.80 ± 0.77^b^	31.12 ± 0.56^c^	29.90 ± 1.16^c^

^a-e^ Different letters within each row indicate significant differences determined by mean ± standard devision (*P* < 0.05).

Treatments: NC, no addition; PC, 0.1 % *l*-ascorbic acid; GF3, 0.03 % GF; GF6, with 0.06 % GF; GF9, 0.09 % GF.

### 3.5 Lipid oxidation of sausages

**Fig. 3:**
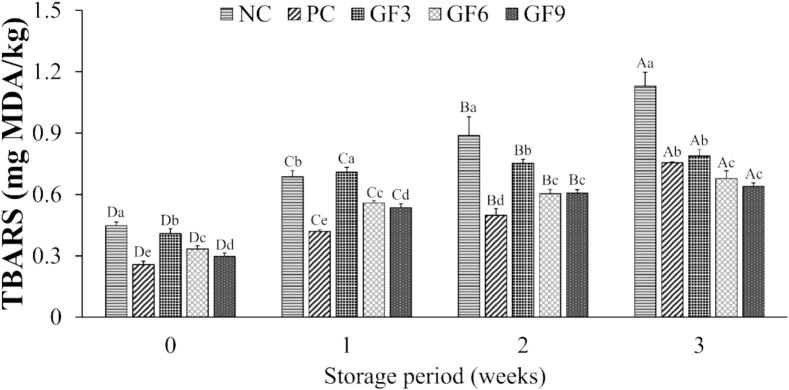
2- thiobarbituric acid reactive substance (TBARS, mg MDA/kg) of emulsion-type pork sausages with *G. frondosa* extract powder during 3 weeks of storage. ^A-D^ Least square means with different letters within each treatment are significantly different (*P* < 0.05). ^a-e^ Least square means with different letters within each week are significantly different (*P* < 0.05).

The lipid oxidation of emulsion-type pork sausages supplemented with various levels of GF during 3 weeks of refrigerated storage is depicted in Fig. 3. In all treatment groups, TBARS values increased over the three-week storage period (*P* < 0.05). The TBARS value of the PC group remained the lowest throughout the storage, whereas the values tended to decrease with increasing levels of GF. The TBARS value of the PC group remained the lowest up to the second week of storage, whereas the values tended to decrease with increasing levels of GF. By the third week, the TBARS values of sausages supplemented with 0.06 % or higher levels of GF (GF6 and GF9) were significantly lower than those of the PC group supplemented with 0.1 % *l*-ascorbic acid (*P* < 0.05). Previous studies have shown reduced TBARS values in patties containing dried *Agaricus bisporus* powder and suppressed lipid oxidation in burger patties supplemented with *Pleurotus ostreatus* (Kido et al., 2023; Tokarczyk et al., 2023; Tom et al., 2018). Lipid oxidation can generally be categorized into autoxidation, enzymatically catalyzed oxidation, and photo- oxidation, with autoxidation being the predominant mechanism responsible for lipid oxidation in meat (Chaijan & Panpipat, 2017). This process is initiated by catalytic compounds such as heat, light, and metal catalysts (e.g., Fe^2+^, Cu^2+^), which promote the formation of reactive oxygen species (ROS), including singlet oxygen (^1^O_2_), superoxide anion (O_2_•^−^), hydrogen peroxide (H_2_O_2_), and hydroxyl radicals (•OH) (Domínguez et al., 2019). These ROS initiate chain reactions that accelerate lipid oxidation in meat (Amaral et al., 2018). In this study, plant-derived phenolic compounds identified in GF were considered to have effectively scavenged these ROS (Manessis et al., 2020). Furthermore, previous research has shown that the hot water extract of *G. frondosa* exhibited superior O_2_•^−^ scavenging activity and Fe^2+^ chelating ability compared to other extraction methods (Yeh et al., 2011), which may have contributed to controlling the lipid oxidation process and reducing TBARS values. These results suggest that GF can suppress lipid oxidation similarly to synthetic antioxidants. Supplementation with at least 0.06 % GF showed antioxidant activity comparable to that of 0.1 % l-ascorbic acid.

### 3.6 Protein oxidation of sausages

**Fig. 4:**
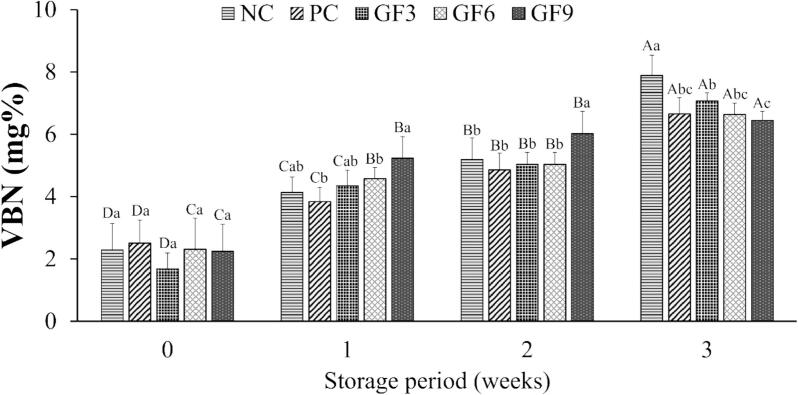
Volatile basic nitrogen (VBN, mg%) of emulsion-type pork sausages with *G. frondosa* extract powder during 3 weeks of storage. ^A-D^ Least square means with different letters within each treatment are significantly different (*P* < 0.05). ^a-c^ Least square means with different letters within each week are significantly different (*P* < 0.05).

The VBN values of emulsion-type pork sausages supplemented with various levels of GF during 3 weeks of refrigerated storage are depicted in Fig. 4. VBN serves as a quality indicator reflecting the freshness of meat products, where higher values signify advancing protein spoilage (Cho et al., 2023). Over the storage period, the VBN values increased significantly (*P* < 0.05), however, no differences were noted among the treatment groups at week 0 (*P* > 0.05). At weeks 1 and 2, GF9 displayed higher VBN values compared to the PC (*P* < 0.05), suggesting a rapid onset of protein degradation. However, by week 3, the VBN values decreased with increasing levels of GF. This indicates that GF9 experienced a rapid increase in VBN during the initial storage period, while GF3 and GF6 exhibited more stable values. These observations align with the study by Park et al. (2023), which observed higher initial VBN values during the first 7 days of storage in beef patties supplemented with Shiitake mushrooms. The rise in VBN is primarily attributed to ammonia formation during storage, resulting from the deamination of amino acids as proteins degrade (Byun JoonSeok et al., 2003). The fruiting body of *G. frondosa* contains aminopeptidase, a protease that selectively hydrolyzes protein peptides (Nishiwaki et al., 2009). This enzyme has been reported to exhibit optimal activity at elevated temperatures and pH levels (Hiwatashi et al., 2004). Therefore, it is plausible that enzymatic activity was preserved during the hot water extraction of GF, and this protease may have contributed to the early increases in VBN by accelerating protein breakdown in the meat. Nevertheless, phenolic compounds may reduce VBN levels either by binding to amino groups through their hydroxyl groups or by exerting antimicrobial effects (Ghollasi-Mood et al., 2016; Maqsood et al., 2013). In addition to its previously reported antioxidant properties, *G. frondosa* has been shown to produce Grifolaone A, a novel furanone-derived terpenoid compound with potent antimicrobial activity (He et al., 2016). Moreover, β-glucan and organic acids in GF have shown broad-spectrum antimicrobial effects in various studies (Pinya et al., 2019; Zhang et al., 2017). Thus, while GF supplementation might make the product more prone to early protein spoilage, it appears to stabilize over time. This trend may reflect a balance between initial enzymatic hydrolysis and the potential stabilizing effects of phenolic compounds. Accordingly, it is necessary to determine the optimal level of GF for use in meat products, and based on the findings of this study, supplementation levels up to 0.06 % GF may provide optimal storage stability. However, further investigation is needed to confirm the long-term impact of GF supplementation on protein stability in meat products.

## 4 Conclusion

This study was undertaken to explore the antioxidant activity of *Grifola frondosa* extract (GF) and its impact on emulsion-type pork sausages. The antioxidant activity of GF, assessed using the DPPH and ABTS free radical scavenging assays, demonstrated a dose-dependent increase. Moreover, high values of TPC and TFC confirmed the presence of bioactive compounds. HPLC analysis further identified key phenolic compounds contributing to these effects, including gallic acid, *p*-hydroxybenzoic acid, vanillic acid, caffeic acid, and *p*-coumaric acid. The incorporation of GF in emulsion-type pork sausages significantly enhanced quality attributes and storage stability. GF3, GF6, and GF9 exhibited higher moisture content compared to the PC. GF elevated the pH, which improved WHC and reduced CL. Furthermore, its antioxidant properties effectively inhibited lipid oxidation and protein degradation, as evidenced by the reduced TBARS and VBN values. However, VBN in GF9 increased rapidly during the early storage period. This increase may be attributable to the proteolytic activity of aminopeptidase present in GF, which could accelerate protein degradation during the initial storage phase. Nonetheless, the antioxidant and antimicrobial properties of GF contributed to the stabilization of VBN levels over time. Among the samples, GF6 demonstrated the most balanced effects, maintaining quality while ensuring storage stability. These findings suggest that GF, particularly at a 0.06 % inclusion level, can serve as a natural additive to improve the water holding capacity and stability emulsion-type pork sausages during refrigerated storage. And future studies should explore the mechanism of proteolytic enzymes in GF and assess its applicability in other meat products and processing conditions.

## CRediT authorship contribution statement

**Soyoung Jang:** Writing – review & editing, Writing – original draft, Software, Methodology, Data curation, Conceptualization. **Sanghun Park:** Validation, Conceptualization. **Gyutae Park:** Data curation, Conceptualization. **Beobmo Ku:** Investigation, Conceptualization. **Minjun Kim:** Investigation, Conceptualization. **Jiwoo Kang:** Software, Conceptualization. **Sungkyun Oh:** Resources. **Hyodong Han:** Resources. **Sohee Kim:** Formal analysis. **Hwayong Lee:** Supervision, Software, Formal analysis, Conceptualization. **Sol-Hee Lee:** Writing – review & editing, Conceptualization. **Jungseok Choi:** Writing – review & editing, Supervision, Project administration, Funding acquisition.

## Declaration of competing interest

The authors declare that they have no known competing financial interests or personal relationships that could have appeared to influence the work reported in this paper.

## Data Availability

Data will be made available on request.
